# Prurigo Pigmentosa Following a Keto Diet and Bariatric Surgery

**DOI:** 10.7759/cureus.24307

**Published:** 2022-04-20

**Authors:** Faris Alkhouri, Samaa Alkhouri, Geoffrey A Potts

**Affiliations:** 1 Dermatology, Oakland University William Beaumont School of Medicine, Rochester, USA; 2 Medicine, Wayne State University School of Medicine, Detroit, USA; 3 Dermatology, Wayne State University School of Medicine, Detroit, USA

**Keywords:** prurigo pigmentosa of nagashima, ketosis, keto rash, bariatric surgery, keto diet, prurigo pigmentosa

## Abstract

Prurigo pigmentosa (PP) is a rare pruritic condition with idiopathic etiology that affects mostly females. It most commonly presents as a rash on the neck and trunk. We report the occurrence of PP in a young woman on two separate occasions; her first episode was following a ketogenic diet and second after undergoing a laparoscopic gastric sleeve surgery. This presents as a unique case because, to our knowledge, PP has only been reported in a small number of cases in the Western world. This presentation could be suggestive of a stronger relationship between PP and the metabolic state of the body. It also outlines the effectiveness of treatment options currently in use for treating PP.

## Introduction

Prurigo pigmentosa (PP) of Nagashima is a rare pruritic condition, typically seen in adolescent girls and young women, with an idiopathic etiology first reported in East Asia. It presents as symmetric, reticulated erythematous papules or vesicles on the neck and trunk with frequent recurrences [1]. Over time, post-inflammatory hyperpigmentation lasting months is common. Dietary changes, friction, sweat, and ketonuria have been noted as associated risk factors [2]. PP was originally reported in Japan and neighboring Asian countries, but current reports from the Western world and the Middle East show that it is more widespread [3,4]. Despite the unknown etiology, treatment using tetracyclines, antibiotics, and dietary changes has been found to provide some degree of relief [2]. We report the occurrence of PP in a young woman, who was of Middle Eastern descent, on two separate occasions. Her first episode was observed after following a ketogenic diet and the second after undergoing a laparoscopic gastric sleeve surgery.

This article was previously presented as a meeting abstract at the 2021 American College of Physicians (ACP) Annual Michigan Chapter Abstract Competition on October 17, 2021.

## Case presentation

**Figure 1:**
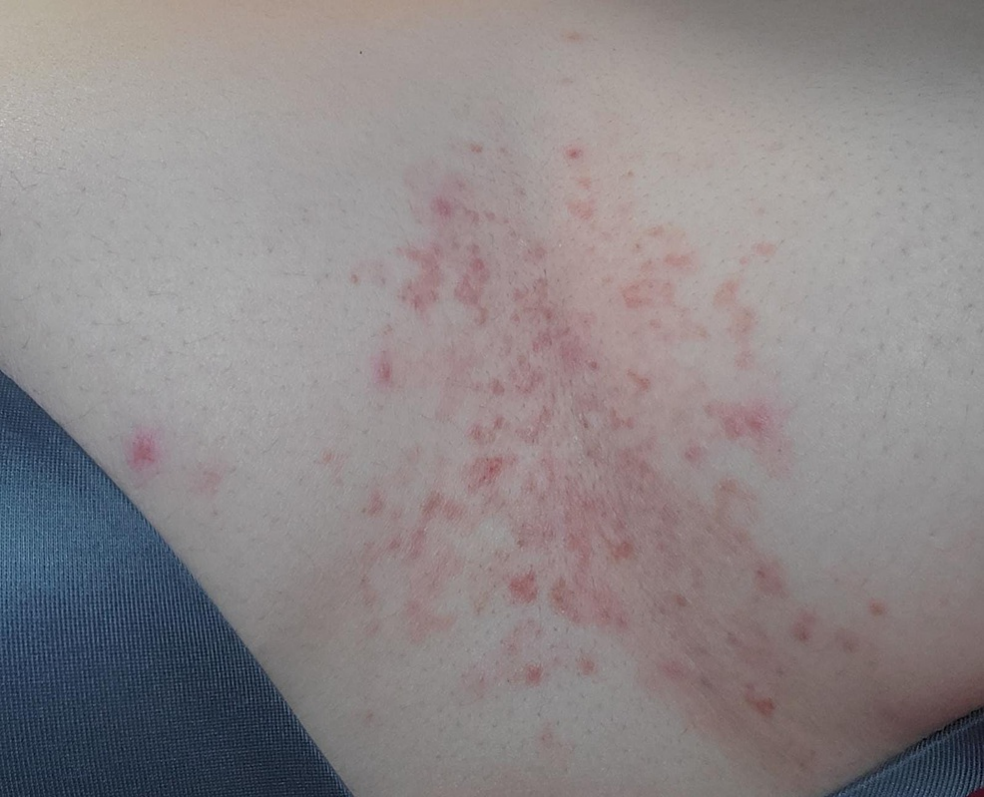
Erythematous papules in the intermammary cleft area

A 25-year-old female of Middle Eastern descent presented with a rash in the intermammary cleft that started three weeks after undergoing gastric sleeve surgery. The rash was pruritic with burning pain only upon scratching. Physical exam and patient history revealed eruptions that had spread over the chest and progressed from small red papules to become coalescent plaques with occasional crusted vesicles (Figure 1). No lesions were found elsewhere on the body. At that time, the patient was instructed to follow the recommended dietary guidelines for post-operative gastric sleeve care that consisted of a high protein intake and low to no carbohydrates as well as vitamin supplementation. The patient attributed the rash to her current diet changes. Notably, she had a similar occurrence and morphology of the eruption one and a half years ago when she attempted the ketogenic diet to lose weight. The rash appeared initially two weeks into the diet and had similar progression as the current presentation, as described by the patient. Five weeks into the ketogenic diet, the patient decided to resort back to her normal, carb-rich diet. Soon after, the rash slowly cleared with no complications over the next month.

In her most recent presentation after gastric sleeve surgery, the patient complained that she was unable to resort back to the carb-rich diet due to the post-operative instructions. Oral minocycline was prescribed to control the rash and a follow-up was scheduled for three weeks. A biopsy was not performed.

Following the three-week period, the inflammatory papules improved after the use of oral minocycline as well as an effort to increase her carbohydrate intake. The rash had subsided, but did not fully resolve, and the patient stopped using minocycline. Despite these measures, when tapering minocycline and lowering the dietary carbohydrate load, the rash recurred. The patient reported later that even after a year since the gastric sleeve surgery, she still gets the rash occasionally when dieting and limiting carbohydrate intake.

## Discussion

There has been a growing association between PP, diet changes, and ketosis. This condition was first described in Japan, and was thought to be limited to the Japanese population [5]. Since then, more cases have been reported in the rest of the world [3,4]. Despite an unknown mechanism of pathophysiology, there have been reports of the occurrence of PP during prolonged fasting, strict diet restrictions, during the period of ketosis following a ketogenic diet, and following bariatric surgery [6,7]. Our patient presents as a unique case because, to our knowledge, this condition has only been reported in a small number of cases in the Western world. Most of the cases reported are either due to ketogenic diet or due to gastric surgeries alone. This case shows the occurrence of PP following both of these conditions in the same individual, which could be suggestive of a stronger relationship between PP and the metabolic state of the body.

Finally, treatment for this case showed its effectiveness as minocycline has been shown to help with PP through its anti-inflammatory mechanism [2,8]. The main contributor, however, that eliminated the rash completely was the incorporation of carbs in her diet over a long period of time, which is what has also been observed in some published reports [9]. This case should serve to raise awareness about this condition as it could be going under-diagnosed due to variable histopathology and overlap with other dermatoses. In patients with poor response to topical corticosteroids, this entity should be entertained in the differential diagnosis. With the constant increase in rates of bariatric surgeries, it is important to watch out for such associations especially during the post-operative period where the patient is more likely to enter ketosis [10].

## Conclusions

This case outlines the presentation of a 25-year-old female who presented with a pruritic rash in the intermammary cleft following an attempt at the ketogenic diet as well as a gastric sleeve surgery. This rash presentation was identified to be prurigo pigmentosa, a rare condition that we have yet to understand the etiology of. However, there seems to be a growing association between PP and the metabolic state of the body, and this case presents further evidence to strengthen this link. The diagnosis of PP should be considered in patients with poor response to topical corticosteroids as well as patients who develop rashes after bariatric surgeries or after starting a keto diet.

## References

[1] Böer A, Misago N, Wolter M, Kiryu H, Wang XD, Ackerman AB (2003). Prurigo pigmentosa: a distinctive inflammatory disease of the skin. Am J Dermatopathol.

[2] Mufti A, Mirali S, Abduelmula A, McDonald KA, Alabdulrazzaq S, Sachdeva M, Yeung J (2021). Clinical manifestations and treatment outcomes in prurigo pigmentosa (Nagashima disease): a systematic review of the literature. JAAD Int.

[3] Hijazi M, Kehdy J, Kibbi AG, Ghosn S (2014). Prurigo pigmentosa: a clinicopathologic study of 4 cases from the Middle East. Am J Dermatopathol.

[4] Jeunon de Sousa Vargas T, Abreu Raposo CM, Lima RB, Sampaio AL, Bordin AB, Jeunon Sousa MA (2017). Prurigo pigmentosa—report of 3 cases from Brazil and literature review. Am J Dermatopathol.

[5] Nagashima M (1978). Prurigo pigmentosa: clinical observations of our 14 cases. J Dermatol.

[6] Alshaya MA, Turkmani MG, Alissa AM (2019). Prurigo pigmentosa following ketogenic diet and bariatric surgery: a growing association. JAAD Case Rep.

[7] Almaani N, Al-Tarawneh AH, Msallam H (2018). Prurigo pigmentosa: a clinicopathological report of three Middle Eastern patients. Case Rep Dermatol Med.

[8] Yang J, Jiao S, Zhang M (2021). Use of minocycline for the treatment of prurigo pigmentosa with intraepidermal vesiculation: a case report. J Int Med Res.

[9] Wong M, Lee E, Wu Y, Lee R (2018). Treatment of prurigo pigmentosa with diet modification: a medical case study. Hawaii J Med Public Health.

[10] (2022). Estimate of bariatric surgery numbers, 2011-2019. https://asmbs.org/resources/estimate-of-bariatric-surgery-numbers.

